# Coalescing traditions—Coalescing people: Community formation in Pannonia after the decline of the Roman Empire

**DOI:** 10.1371/journal.pone.0231760

**Published:** 2020-04-29

**Authors:** Corina Knipper, István Koncz, János Gábor Ódor, Balázs Gusztáv Mende, Zsófia Rácz, Sandra Kraus, Robin van Gyseghem, Ronny Friedrich, Tivadar Vida

**Affiliations:** 1 Curt-Engelhorn-Center Archaeometery gGmbH, Mannheim, Germany; 2 Institute of Archaeological Sciences, ELTE–Eötvös Loránd University, Budapest, Hungary; 3 Wosinsky Mór Museum, Szekszárd, Hungary; 4 Research Centre for the Humanities, Hungarian Academy of Sciences, Budapest, Hungary; University at Buffalo - The State University of New York, UNITED STATES

## Abstract

The decline of the Roman rule caused significant political instability and led to the emergence of various ‘Barbarian’ powers. While the names of the involved groups appeared in written sources, it is largely unknown how these changes affected the daily lives of the people during the 5^th^ century AD. Did late Roman traditions persist, did new customs emerge, and did both amalgamate into new cultural expressions? A prime area to investigate these population and settlement historical changes is the Carpathian Basin (Hungary). Particularly, we studied archaeological and anthropological evidence, as well as radiogenic and stable isotope ratios of strontium, carbon, and nitrogen of human remains from 96 graves at the cemetery of Mözs-Icsei dűlő. Integrated data analysis suggests that most members of the founder generation at the site exhibited burial practises of late Antique traditions, even though they were heterogeneous regarding their places of origin and dietary habits. Furthermore, the isotope data disclosed a nonlocal group of people with similar dietary habits. According to the archaeological evidence, they joined the community a few decades after the founder generation and followed mainly foreign traditions with artificial skull modification as their most prominent characteristic. Moreover, individuals with modified skulls and late Antique grave attributes attest to deliberate cultural amalgamation, whereas burials of largely different isotope ratios underline the recipient habitus of the community. The integration of archaeological and bioarchaeological information at the individual level discloses the complex coalescence of people and traditions during the 5^th^ century.

## Introduction

The decades before and after the gradual decline of the Roman rule in Pannonia were politically unstable. From the last decades of the 4^th^ century AD onwards, population groups pushed by or fleeing from the Huns arrived continuously to the Carpathian Basin [1, 2]. Part of them settled down and the Roman administration attempted to integrate these groups through *feoderati* treaties. The cohabitation and later the amalgamation of locals and foreign, non-Roman groups lead to a continuous cultural transformation during the 5^th^ century that affected both lifestyle and material culture [2]. After the appearance of the Huns in Pannonia at the beginning of the second third of the 5^th^ century and the abandonment of the area by the Romans (433 AD), the population decreased and the settlement structure changed drastically [3]. Communities fled to the western provinces with the promise of safety, while others sought refuge in forts and cities looking for protection. The political, social, and economic roles of the cities disappeared or changed with the diminishing of the Roman administration in the region. While they probably still maintained their local importance for some time, both their size and population decreased seriously [2, 4, 5]. The newly arriving groups also founded rural settlements often in connection to the former Roman infrastructure, such as roads and fortified places [6]. After the collapse of the Hunnic power in the middle of the 5^th^ century, various regional polities of different Barbarian groups, such as Goths, Suebi, Rugii, Alans etc. emerged. Their disagreements, competing interests, and occasional wars lead to different community-level realignments and renewed changes in the settlement network.

Profound changes also affected the burial practises and material culture. The late Roman (2^nd^ to 4^th^ cent.) cemeteries were gradually abandoned and single inhumations as well as small burial groups appeared. The newly founded cemeteries of the middle of the 5^th^ century document a mosaic-like structure of population groups, cultural amalgamation and interregional connections. The sites exhibit both evidence for late Roman traditions and features that did not occur in the late Roman cemeteries in Pannonia, including a new burial representation of high-status women [7, 8]. Among the newly attested attributes are burials with side niches and artificially deformed skulls that probably arrived with representatives of non-local populations. Both forms and furnishings of the graves as well as the skeletal remains provide direct and individual evidence for the superordinate processes of their time.

This study focusses on the cemetery of Mözs-Icsei dűlő (Hungary), which is one of the largest, fully excavated cemeteries of the 5^th^ century in the former Roman province of *Pannonia Valeria*. Reflecting the period of profound cultural upheaval, the integration of archaeological, anthropological, and multi-isotopic (^87^Sr/^86^Sr, δ^18^O, δ^13^C, δ^15^N) information sheds direct light on group dynamics, dietary habits, and cultural coalescence. While written sources sketch the general historical and political framework, it is largely unknown how the fundamental changes affected the actual lives of the people. How did new communities emerge from groups of heterogeneous origins? Is it possible to define migrating groups, and if so, how did the migrations effect the every-day life of these communities? To which extent did provincial ‘Roman’ customs continue, and how did practises of the newly arriving non-Roman groups manifest?

## The cemetery of Mözs-Icsei dűlő

**Fig 1:**
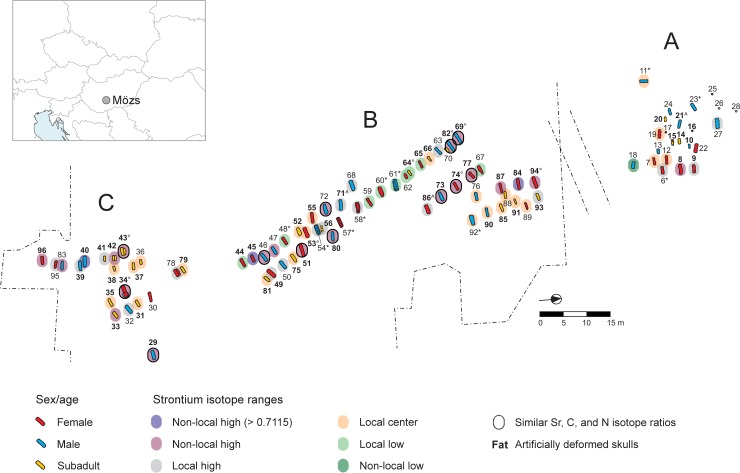
Map of the cemetery of Mözs-Icsei dűlő and its location in present-day Hungary. The cemetery consists of three burial groups (A, B, C). Bold burial numbers highlight inhumations with artificially modified skulls. Symbols after the burial number indicate: *: individuals with indication of being buried in the 2^nd^ quarter of the 5^th^ century AD (early burials); °: individuals with indication of being buried in the 3^rd^ quarter of the 5^th^ century AD (late burials); and ^: individuals with indication of elements of both local and foreign cultural traditions.

The cemetery of Mözs-Icsei dűlő (46°22'54.26"N 18°44'5.44"E) was established right after the decline of the Roman Empire in the second quarter of the 5^th^ century AD. The first 28 graves were excavated by Ágnes Salamon in 1961 [9], while János Gábor Ódor investigated 68 additional graves between 1995 and 1996 [10]. The site is located in the former Province of *Pannonia Valeria* (today Transdanubia, Western Hungary) not far from a backwater of the Danube (Tolnai-Duna), around 10 km East of the modern course of the river (Fig 1). It is situated at the meeting point of three geographical regions: the Sárköz to the South, a plain with abandoned channels and loops of regulated rivers; the Mezőföld to the North; and the Tolna highlands to the West, which are articulated by small river valleys. The site lies on the south-eastern slope of a sand hill, in a landscape that is dominated by loess and other Pleistocene sediments like the rest of the Sárköz.

The 96 graves of the fully excavated cemetery belong to three burial groups. The northern group (A) consists of the 28 graves excavated by Ágnes Salamon. Group B, in the middle, 30–40 m south of group A, is the largest and contains 48 graves in multiple rows. The southern burial group (C), another 15 m to the South, comprises 20 less well-organised inhumations in at least two rows. All burials had West-East orientation except for Grave 11, which was oriented to the North and separated from Group A.

Forty of the 89 burials that could be evaluated in this regard contained dress accessories, such as belt buckles or different types of jewellery, tools for everyday life (e.g. knives) or items, such as combs, that were either dress accessories or tools (grave goods are listed in S1A Table). While most artefacts including two-sided bone combs, polyhedric earrings, iron brooches with inverted foot, iron buckles etc. are not suitable for exact dating within the 5^th^ century, there are some types that define the chronological framework of the cemetery. The jar and the bronze (?) buckles decorated with bird heads and almandine inlays from Grave 11 as well as the bone comb with an arching back, decorated with horse heads, date to the first half of the 5^th^ century. Brick structure graves constructed of Roman *tubuli* and *tegulae* indirectly suggest a similar dating. They are present in cemeteries of the first half of the 5^th^ century and no longer characteristic in the second half of it. Two pairs of the so-called Bakodpuszta-type brooches (Graves 53 and 64), a silver, axe-shaped pendant and a silver, crescent-shaped pendant from Grave 43 as well as the one-sided comb from Grave 34 suggest that the site remained in use even after the middle of the 5^th^ century [2, 11, 12]. Based on the available archaeological data, the cemetery was in use for at least two generations between the second and third quarter of the 5^th^ century AD. Grave forms and grave goods suggest minor chronological differences among the three burial groups. They indicate that Group A and B were founded around 430/440 AD. In Group B, the earliest burials cluster in the middle of the main grave row with a series of brick structure graves [54, 57, 58, 60, 61], while the burials further out or at the ends of the rows contained chronologically later artefact types [64, 48, 69, 82] (Fig 1). There are at least three burial rows, which may have originated from multiple early cores. Group C was founded one or two decades later. Artefacts dated to the second half of the 5^th^ century (such as Bakodpuszta type brooches, single-sided combs, lunula- and axe-shaped pendants and early forms of earrings with basket-shaped pendants, etc.) only occurred in Group B and C (S1A Table). This suggests that Group A was abandoned when Group C was founded or slightly after that, and Group B and C remained in use until the abandonment of the site.

**Fig 2:**
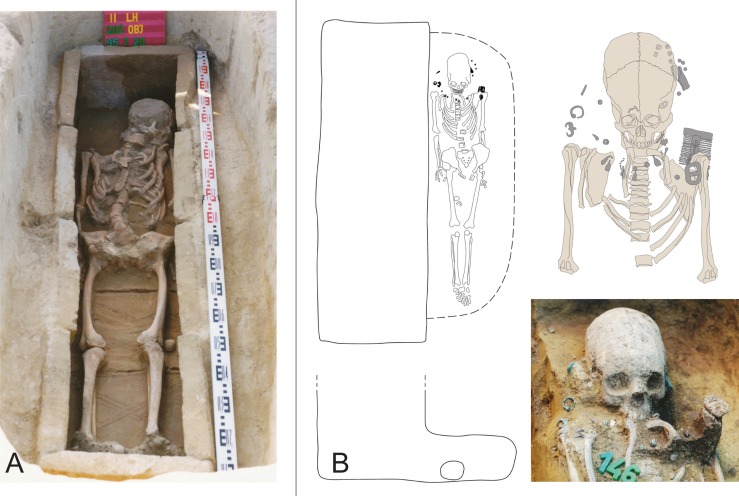
Examples of burials from the cemetery of Mözs-Icsei dűlő. A: The brick-lined burial of Grave 54 represents late Antique traditions, which prevailed among the supposed founder generation of the cemetery. B: Grave 43, a niche burial of a child, probably a member of a later joining group, with an artificially deformed skull and a rich inventory dating to the third quarter of the 5^th^ century (see discussion below).

With its almost a hundred burials of the early 5^th^ century, the cemetery of Mözs is so far unique in the territory of Pannonia. Analogies are only known from Moesia Superior, in the area of the former province capital of Viminacium [13]. Grave forms, such as brick structure graves, and grave goods, such as earrings with a basket-shaped pendant, were typical at large late Roman cemeteries of the 3^rd^ and 4^th^ centuries and are referred to as elements of late Roman/Antique traditions. Objects, such as iron buckles with damascening, Bakodpuszta-type brooches, or certain kinds of combs root in Roman traditions, but have been modified over time. In contrast, features, such as graves with a side niche and artificially deformed skulls (Fig 2) arrived with representatives of non-local populations, and are considered elements of a foreign–most probably non-Roman–tradition [14]. Graves with a side niche (n = 3) were restricted to the southern burial group (Group C) (S1A Table, Fig 1). Among the ledge graves, four occurred in the middle group (B) and one in the north (A). Brick structures were found in the northern (A; n = 4) and middle group (B; n = 6). The rest of the burials were simple rectangular pits. Four graves showed traces of a coffin. One of them belonged to group C, and three to group B.

The evidence for both Roman and foreign cultural traditions at Mözs provoked different classifications and associations of the site. It has been considered to represent the Szabadbattyán type of cemeteries, which refers to sites that were founded by immigrants during the middle of the 5^th^ century. Alternative interpretations included a burial site of Hun-Gothic-Alan *foederati*, a Roman cemetery showing ‘Barbarian’ influence, a site with strong East Germanic character, or a cemetery of a ‘Barbarian *gentes*’ living a sedentary life in Transdanubia [8, 14–16].

A contemporary settlement was located on a hill, about 100 meters west of the cemetery and excavated between 1995 and 1999. Altogether 15 sunken-featured buildings and 56 pits were unearthed [14]. The majority of the finds were pottery with strong influences of late Roman tradition. The fragments of a Murga-type jug, as it occurred in grave 11 at the cemetery, and spouted vessels clearly date the settlement to the 5^th^ century. Moreover, observations including analogies of the wheel-made bowl of grave 64, double-sided combs and bronze tweezers indicate a direct relation between the cemetery and the settlement. Finally, the close proximity of the sites and the lack of any other nearby features of the 5^th^-century indirectly support the connection between the settlement and the cemetery.

## Methodological background of physical anthropological and multi-isotope analyses

### Physical anthropology

This study followed an interdisciplinary approach and integrated the archaeological evaluation of the cemetery with the results of physical anthropological as well as stable and radiogenic isotope analyses.

The age at death was estimated using well-established methods [17–22]. Age categories include infans I (0–6 years), infans II (7–14 years), juvenile (15–22 years), adultus (23–39 years), maturus (40–60 years), and senilis (> 60 years) with intermediate forms (adultus-maturus and maturus-senilis). Sex determination was carried out by using 22 characteristics of the skeleton [23]. In the case of juveniles, sex estimations are based on the overall robustness of the skeleton, selected criteria of the pelvic bones, such as the insicura ischiatica major, the presence of the sulcus praeuricaularis, the subpubic angle and the relative width of the sacrum. In addition, major characteristics of sex differentiation of the skulls were assessed. Specific observations for the respective individuals are listed in S1A Table.

Special emphasis was put on the documentation of artificial modifications of the skulls, which is a main characteristic of the burials at the site. The final shape of an anthropogenically modified skull is determined by a combination of both, the deformation technique [24] and the polygenic determination of the shape and the dimensions of the skull, whose development terminates by the end of childhood. In addition, external factors, such as defects connected to birth or certain hereditary or acquired diseases can affect the morphological character of the phenotype. Furthermore, *post mortem* distortions during burial may cause deformations. Most often, they occur among young children, but differ in their directions from artificial deformations.

There are several methods for measuring the degree of deformation, among which the determination of so-called deformation indices are the most established (e.g. *Oetteking-Ginzburg-Zirov index*, [25–27]. Referring to commonly used anthropometric points, they indicate the extent of changes of the dimensions of the cranial vault. The evaluation of deformation indices is based on the premise that there are fixed points of the vault (primarily at the skull base) that stay unaffected by the deformation process. However, this premise cannot be proven by solid statistics, and the distinction between congenital and artificial cranial deformation remains often unclear [28, 29]. Most deformation indices are only informative in case of highly deformed skulls of adult individuals, where the deformation results in a major change in the dimensions of the neurocranium. Therefore, characteristic recessions on the cranial bones, which result from various bandage techniques, were additionally registered as indications of artificial deformation. These traces are also visible on fragmented or not fully developed skulls of children with incompletely fused sutures and indicate artificial deformation where detailed measurements are impossible.

### Strontium isotope analysis

Strontium (^87^Sr/^86^Sr) isotope analysis of tooth enamel aimed at recognising individuals of different origins. The method is frequently applied to identify human individuals or animals who grew up in an area with geological conditions that differed from those of the location where their mortuary remains were found. The distinction among local and non-local individuals provides the basis to evaluate the importance and nature of human mobility and to address questions regarding residential systems and mechanisms behind the distribution of economic and cultural traditions [30–34].

Strontium is a trace element that frequently occurs in rocks. It has four stable isotopes (^84^Sr, ^86^Sr, ^87^Sr und ^88^Sr) of which ^87^Sr is partly radiogenic and results from radioactive decay of ^87^Rb (Rubidium). This process causes the fraction of ^87^Sr–expressed as ^87^Sr/^86^Sr ratio–to vary among geological units depending on their original rubidium contents and ages [35–37]. Weathering of the bedrock releases strontium into soils and groundwater, from where it is taken up by plants and passed on via the food chain to animals and humans. Isotope fractionation during this process is negligible and corrected during data processing [38]. Therefore, the Sr isotope ratios of foodstuffs reflect the geological conditions at the localities from which they originated. In humans and animals, most of the strontium is incorporated in the inorganic fraction (hydroxyapatite) of teeth and bones, in which the trace element substitutes for calcium. Enamel of the tooth crowns is the most informative sample material. While some of the deciduous teeth already start forming in utero, the crowns of the permanent dentition mineralize largely between birth and adolescence [39] and remain afterwards unchanged [40]. Enamel is also a very hard and dense material and therefore more resistant to diagenetic alteration than dentine or bone [41, 42]. The classification of ^87^Sr/^86^Sr ratios as non-local requires comparative data that characterize the isotope ratios of the biologically available strontium. Samples to establish Sr isotope baselines can include enamel of supposedly locally fed animals from archaeological contexts, modern water or vegetation as well as archaeological bones [43–47].

Here, we evaluated the data distribution of several independent datasets in comparison to each other. First, we used enamel of contemporaneous domestic pigs and sheep/goats from the settlement of Mözs. Strictly, it cannot be excluded that some of the animals were introduced by their potentially mobile owners so that some of the data may represent an area beyond possible pastures within a few kilometres around the site [48, 49]. In addition, we considered chronologically older (Neolithic) human teeth and bones from four sites in up to 20 km distance from Mözs. Earlier studies identified comparatively small numbers of non-local individuals among Neolithic and Copper Age populations in the Carpathian Basin and confirmed a largely sedentary lifestyle of these groups [50]. Even though, there is an uncertainty regarding the local origin of each of the human individuals, the empirical evaluation of these data follows the assumption that major geological conditions have not changed between the Neolithic and the Migration period and an accumulation of data in a certain range indicates the ‘local human range’ of the bio-available strontium [51]. Among the Neolithic samples, bones are probably the most robust indicator of local Sr isotope values at a given site, because they are not only remodelled continuously in lifetime, but also susceptible to diagenesis and incorporate labile strontium of the local burial environment [41]. Furthermore, we considered the distribution of the human dataset from the cemetery of Mözs itself, particularly, deciduous and permanent teeth of individuals who died before reaching 14 years of age (age groups infans I and infans II). Following Burton and Price [51], we assumed empirically that the major mode of the data indicates the isotope composition of the locally bio-available strontium originating from the agriculturally exploited land within a limited radius around the site. This approach sorts the respective data from the smallest to the largest value and identifies plateaus in the data distribution that are separated by slope breaks. We concentrated on children’s teeth because they contain strontium that was stored a few months or years prior to death of the respective individuals [39, 40]. The short time span between the incorporation of the strontium and the premature death of the individuals, increase the probability of the trace element for being of local origin. Several larger, previously published series of Sr isotope data confirm significantly less variable ^87^Sr/^86^Sr ratios among the teeth of children than among those of adult individuals [32, 49, 52–54]. Alt et al. [49] and Knipper et al. [52] discussed their value as representatives of the strontium that originated from a dietary catchment of a few kilometres radius of a given site extensively. Because strontium isotopes do not fractionate remarkably during metabolic processes, systematic differences between deciduous teeth, that start forming in utero, and permanent teeth, that start forming *postpartum*, are neither expected nor found in empirical comparisons [52]. Nevertheless, infants’ teeth may occasionally reflect non-local isotope ratios, either due to enamel formation *in utero* of a non-local woman who moved during pregnancy, or due to movement of a child with its family or for other reasons for child mobility. Similar to the suggestion by Burton and Price [51] for complete datasets, outliers or slope breaks among the isotope ratios of teeth of children identify potentially non-local individuals and allow excluding them from the definition of the local range. Finally, we considered previously published human, animal and environmental data from archaeological sites throughout the Carpathian Basin. In summary, the empirical combination of different independent datasets, including patterns of internal data distribution, is a promising way for the assessment of the local range of strontium isotope variation.

### Carbon and nitrogen isotope analysis

Stable carbon and nitrogen isotope compositions (δ^13^C and δ^15^N) of bone collagen can discern differences regarding dietary habits within and among burial contexts [55–58], but also reveal agricultural practices and the habitats in which staple crops were grown and animals grazed [59–61]. C_3_ plants with δ^13^C values between -35 and -22 ‰ vs. V-PDB [62] dominate the central European vegetation. They include cereals such as barley and wheat as well as many other plants that typically form the base of food webs in this region. In contrast, millet (*Panicum miliaceum*) follows the C_4_ photosynthetic pathway and exhibits higher δ^13^C of between -12.7 and -11.4 ‰ [63]. Within the spectrum of C_3_ plants, variation occurs due to differences in growing conditions in more canopied or in open habitats and due to different levels of humidity [64–66]. Due to isotope fractionation, δ^13^C values of herbivore collagen are about 5 ‰ higher than those of their plant forage [67], while the difference in collagen δ^13^C between representatives of two adjacent trophic levels is 0.8 to 1.3 ‰ (average about 1 ‰) [68–70]. Trophic level isotopic enrichment also causes considerable variation in nitrogen isotope ratios and provides the basis for estimating the proportions of meat and dairy in the human diet. Delta^15^N values increase by about 3 to 5 ‰ vs. AIR [71] or even by about 6 ‰ per trophic level [72]. Manuring of arable land also raises δ^15^N values of staple food plants [61, 73] and may obscure data interpretation regarding the proportions of different foodstuffs in the human diet.

### Previous studies

Previous strontium isotope studies on human mobility in the Carpathian Basin concentrated on the Neolithic and Copper Age [50, 74–77], the Bronze Age [78] as well as the Migration Period [48, 49, 79, 80]. Hakenbeck et al. [48] investigated samples of five cemeteries of the 5^th^ century AD, including those of the burials from group A at Mözs. In Szólád at Lake Balaton, the combination of strontium and light stable isotope analyses revealed human mobility and dietary habits during the Lombard period of the 6^th^ century AD [49].

## Material and laboratory methods

This study produced ^87^Sr/^86^Sr ratios of tooth enamel as well as δ^13^C and δ^15^N values of bone collagen of 68 individuals from burial groups B (n = 48) and C (n = 20) at the cemetery of Mözs (S1A Table). The newly analysed skeletons and animal remains are stored at the Wosinsky Mór Museum at Szekszárd and were sampled with permission by János Gábor Ódor (director of the museum and excavator of the site) and Balázs G. Mende (conductor of the primary anthropological analysis). The inventory numbers of the skeletons (97.1.1–97.1.68) are listed in S1A Table.

Permanent second molars were preferred for Sr isotope analysis. First and third molars or deciduous teeth served as alternatives, if second molars were not yet formed or not available. Because three burials did not have any teeth preserved, 65 teeth were sampled in the course of this study. Their ^87^Sr/^86^Sr ratios were evaluated together with ten Sr isotope readings of second molars from burials of group A previously published by Hakenbeck et al. [48]. C and N isotope analysis was conducted on collagen from ribs or long bones (humerus, femur, tibia, or fibula), that represent all of the 68 individuals from burial groups B and C. The data were combined with 11 carbon and nitrogen isotope readings from burial group A previously published by Hakenbeck et al. [48]. The isotope readings for burial group A represent 14 individuals, of which seven were tested for strontium as well as for the carbon and nitrogen (S1A Table).

Altogether, the study comprised data of 82 individuals. Among them were 12 individuals of the age groups infans I (0–6 years) and nine of the age group infans II (7–14 years). We evaluated the data for an infans II-juvenile individual and a probable infant with the infans II group. For certain aspects of data discussion, the individuals of the infans I and infans II categories were evaluated together as ‘children’ (n = 21). Five juveniles (15–22 years) and two individuals of juvenile to adult age were assigned to female (n = 6) or male (n = 1) sex. Their isotope and archaeological data were evaluated along with those of the adults, who comprised 33 females, 27 males, and 1 individual of unknown sex and age.

Enamel of five pigs and a cattle tooth from the contemporaneous settlement were sampled as potential representatives of the isotopic composition of the biologically available strontium near the site (S1B Table). Especially for the pigs, it seems likely that they foraged on resources from within a few kilometres of the settlement and cemetery. However, domestic animals reflect human subsistence and non-local origins cannot be ruled out completely for any single individual.

For comparison, we also considered enamel and bone samples of 16 human burials and enamel samples of pigs or sheep/goats from four Neolithic sites in up to 20 km distance from Mözs (S1C Table). The sites represent geologic and environmental conditions that are very similar to the site of Mözs and have not changed substantially between the Neolithic and the Migration period. Despite, again, we cannot rule out a non-local origin for any of these specimens, especially not for the human teeth, the overall distribution of the data should reflect the biologically available strontium in the Sárköz and Mezőföld area.

Thirty-six bones of sheep/goat, cattle, pig, dog, and poultry from the settlement of Mözs gave estimates of the C and N isotope ratios of herbivores and omnivores. They include 14 new analyses (S1B Table) and 22 previously published data [48].

Strontium isotope analysis was carried out at the Curt Engelhorn Center Archaeometry, Mannheim, Germany and followed previously described methods [32, 81, 82]. Enamel samples were cut and mechanically cleaned, ground, pre-treated with 0.1 M acetic acid buffered with Li-acetate (pH 4.5) in an ultrasonic bath, rinsed, and ashed. Sr separation with Eichrome Sr-Spec resin was carried out under clean-lab conditions. Sr concentrations were determined by Quadrupole-Inductively Coupled Plasma-Mass Spectrometry (Q-ICP-MS), and the isotope ratios by High-Resolution Multi Collector-ICP-MS (Neptune). Raw data were corrected according to the exponential mass fractionation law to ^88^Sr/^86^Sr = 8.375209. Blank values were lower than 10 pg Sr during the whole clean lab procedure. The NBS 987 and Eimer & Amend (E & A) standards run along with the human samples yielded ^87^Sr/^86^Sr ratios of 0.71023 ± 0.00005, 2 σ; n = 21 and 0.70802 ± 0.00005, 2 σ; n = 23, respectively.

Collagen extraction followed the method laid out by Longin [83] with modifications as described in Knipper et al. [84]. Mechanically cleaned bone samples were demineralized in 0.5 N HCl, rinsed, reacted with 0.1 M NaOH, rinsed again, gelatinized, filtered with EZEE filter separators, frozen, and lyophilized. C and N contents and the stable isotopic compositions were determined in triplicates using a Thermo Flash 2000 Organic Elemental Analyzer coupled to a Thermo Finnigan Mat 253 mass spectrometer at the Department of Applied and Analytical Palaeontology, Institute of Geosciences at the University Mainz or a vario PYRO cube CNSOH elemental analyzer (Elementar) and a precisION isotope ratios mass spectrometer (Isoprime) at the Curt Engelhorn Center for Archaeometry Mannheim. The raw data were calibrated against the international Standards USGS 40 and USGS 41 using an off-line two-point calibration [85] or the IonOS software for stable isotope analysis. Interspersed quality control standards gave the δ^13^C and δ^15^N values listed in S1D Table.

The data were evaluated using descriptive statistics and graphics in Microsoft Excel and SigmaPlot 14.0. The normality of data distributions within groups was tested by the Shapiro-Wilk test. The Mann-Whitney Rank Sum test was applied for pairwise comparisons of datasets that did not follow a normal distribution. Pairs of normally distributed datasets were tested for equal variances using the Brown-Forsythe test. In case of equal variances, the significance of differences was determined using the Student’s t-test, whereas the Welch’s t-test was considered in cases of different variances. Multiple datasets were compared using One Way ANOVA (normal data distribution) or ANOVA on Ranks (not normally distributed data). The statistical significance level was p < 0.05 in all applied tests.

## Results

### Physical anthropology and artificial skull deformations

The 28 burials from Group A have been previously investigated [9]. Due to bad preservation, only 19 produced sufficient anthropological data. Balázs Gusztáv Mende investigated the skeletons of the groups B and C. These 68 burials included 26 adult females/possible females and 21 adult males (S1A Table). Individuals of the age groups adultus/adultus-maturus (n = 21) and maturus/maturus-senilis (n = 19) occurred in similar frequencies, whereas there were only two individuals of the oldest age group (senilis). Among the 21 children, twelve were assigned to the age group infans I (0–6 years), seven to the age group infans II (7–14 years), and one could not be further categorized. Three individuals were of a juvenile age (15–22 years). Both anthropological investigations combined identified 87 individuals: 29 females, 26 males, four juveniles, 27 children (infans I and II combined) and one individual of unkown age and sex. All three burial groups contained both adult males, females, and children (S1A Table). With 50% (n = 10) of the individuals, children were most frequent in burial group C, while 31.6% (n = 6) of the graves of group A and 22.9% (n = 11) of the graves of group B yielded remains of subadult individuals.

**Fig 3:**
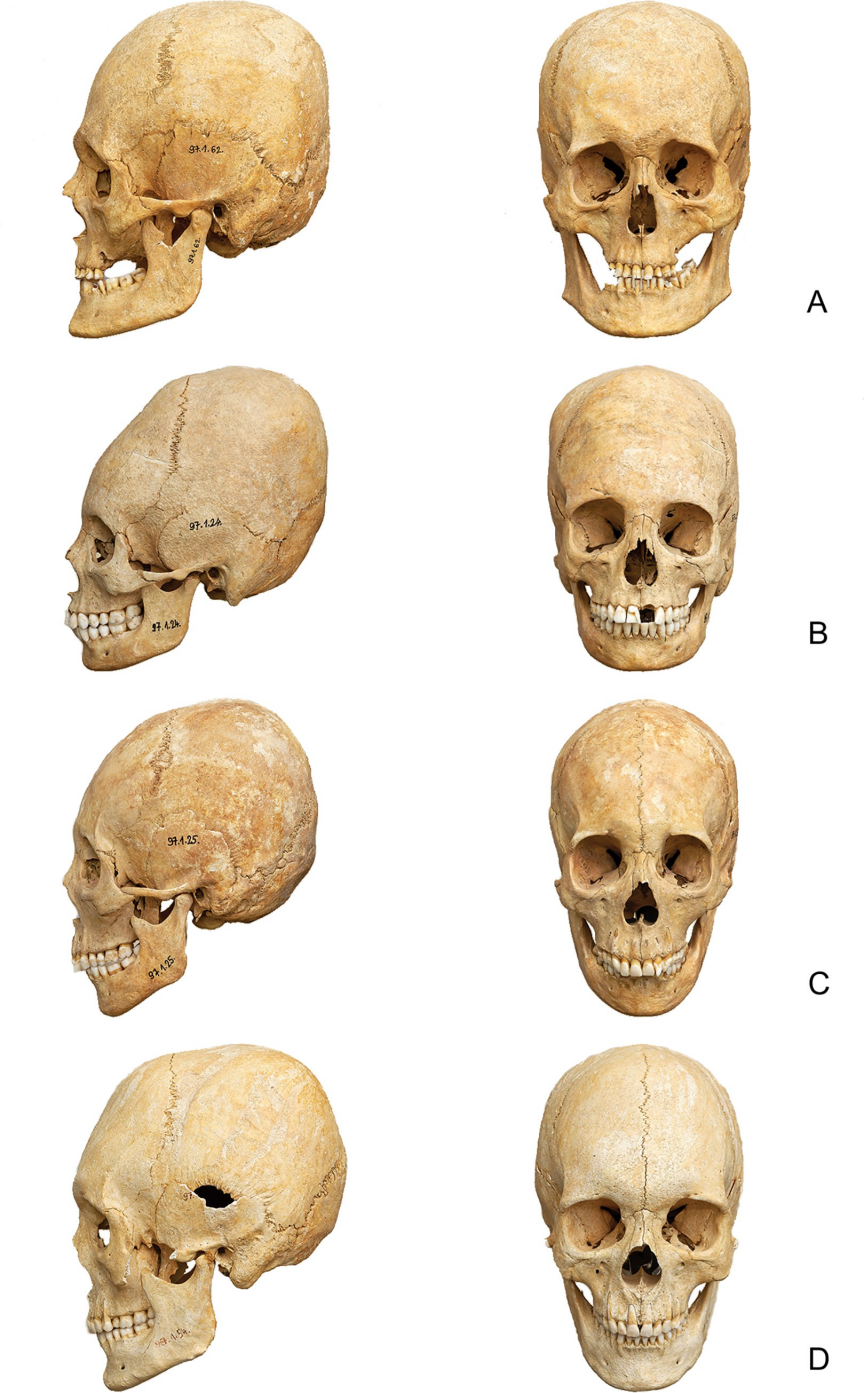
Examples of artificially modified skulls at the cemetery of Mözs. A: Variant I: traces of circular binding causing a strong upward deformation (grave 90), B: Variant II: traces of binding on the forehead causing a strong backward-upward deformation (grave 52), C: Variant III: medium degree of circular deformation, mostly backwards (grave 53), and D: Variant IV: slight deformation due to cross-bandages (grave 82).

Fifty-one individuals, including adult males, females, and children had artificially deformed skulls, making Mözs-Icsei dűlő one of the largest concentrations of this phenomenon in the Carpathian Basin. Five individuals with artificially deformed skulls from group A are not listed in S1A Table, because they were excluded from sampling for isotope analysis [48]. Recessions on the surface of the skulls that resulted from the application of bandages were the most remarkable evidence for anthropogenic deformations. Based on the locations and directions of the grooves, four different variants of deformations were distinguished and point to different banding techniques (S1A Table, Fig 3). Seven skulls [Graves 29, 31, 33, 39, 75, 85 and 90] showed traces of circular binding with cloth bands, which resulted in a strong upward deformation (Variant I). Seven other cases [Graves 34, 35, 43, 44, 52, 77 and 93] had traces of strong binding on the forehead (frontal bone) with deformation backwards-upwards (Variant II). Eleven skulls [Graves 45, 53, 65, 66, 74, 79, 80, 81, 86, 94 and 96] showed a medium degree of circular, mostly backward deformation (Variant III), while six [Graves 40, 51, 69, 71, 73 and 82] were slightly deformed with the help of cross-bandages (Variant IV). In several instances, the recessions that formed typical patterns of some of the binding techniques were already distinguishable among the skulls of children. For instance, both the adult female of grave 34 and the infans II child of grave 43 exhibited a very similar backward-upward elongation of their skulls, which is characteristic for deformation variant II (cf. S1 Fig). In certain cases, traces of both artificial (*ante mortem*) and secondary (*post mortem*) deformations occurred on the same skull. A representative example is grave 85 (infans I), where traces of a circular bandage are clearly visible, that led to a remarkable change in the maximum length of the skull. In addition, deformation of the neurocranium along the sagittal plane (maximum width) is the result of *post mortem* effects (cf. S1 Fig).

Individuals with artificially deformed skulls occurred throughout the cemetery and comprised 31.6% (n = 6) of the burials in Group A, 64.6% of the individuals in Group B (n = 31) and 70% of the individuals in Group C (n = 14).

### Strontium isotope ratios

**Fig 4:**
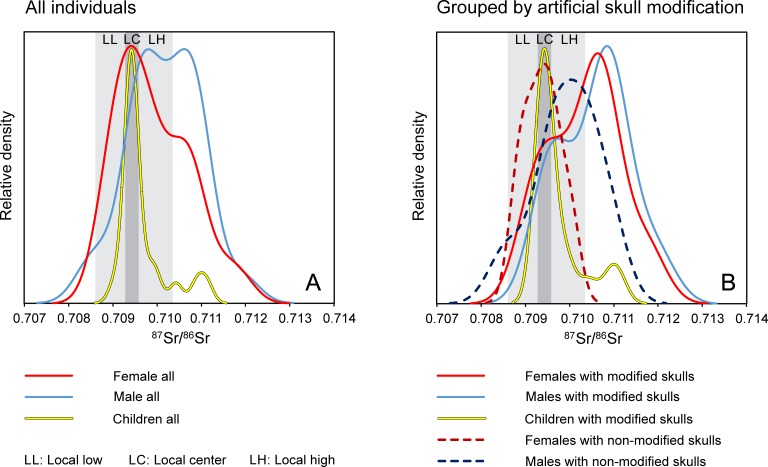
Kernel density curves of the ^87^Sr/^86^Sr ratio distribution. A) Differentiation according to sex and age; B) Differentiation according to sex and age as well as presence or absence of artificial skull modifications.

**Fig 5:**
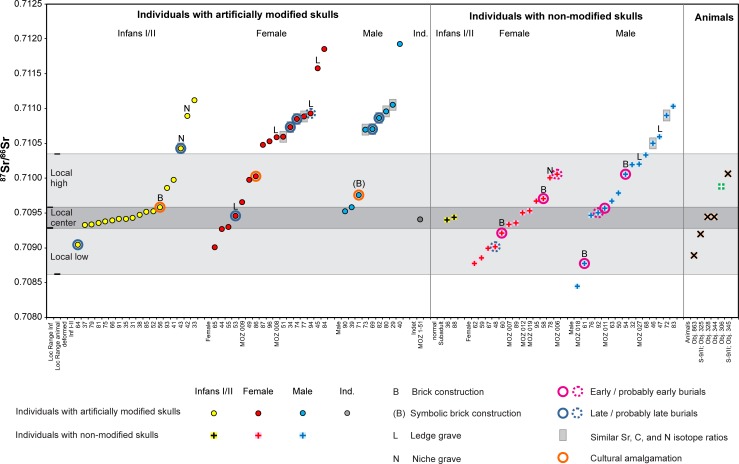
Distribution of the ^87^Sr/^86^Sr ratios of enamel of the burials of the cemetery of Mözs. The individuals are grouped according to the presence or absence of artificial skull modifications and within these groups by subadult individuals as well as adult females and males. Within each group, the data are sorted from lower to higher values. See text for definition of the local strontium isotope ranges.

**Fig 6:**
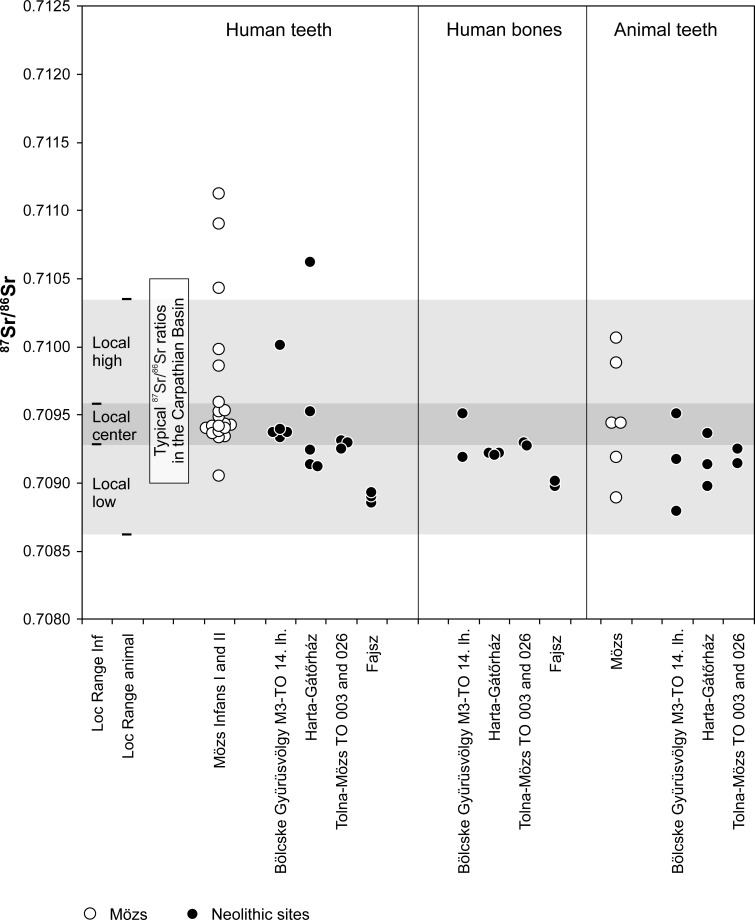
Estimations of the data ranges of the isotope composition of the biologically available strontium in the Carpathian Basin and at the site of Mözs. The ranges ‘local center’, ‘local high’, and ‘local low’ are based on the distribution of the ^87^Sr/^86^Sr ratios of animal teeth from the 5^th^ century settlement of Mözs and the enamel samples of the children from the cemetery (infans I and II combined; explanation see text). The ^87^Sr/^86^Sr ratios of the human enamel, human bones, and animal enamel from four Neolithic sites in up to 20 km distance are plotted for comparison. The light box summarizes typical ^87^Sr/^86^Sr ratios in the Carpathian Basin (cf. S2 Fig).

The strontium isotope ratios of the human teeth from the cemetery of Mözs varied widely between 0.70844 and 0.71193 (S1A Table). The children of the age groups infans I (0–6 years) and infans II (7–14 years) revealed values between 0.70905 and 0.71112 with 14 of the 20 samples (70%) concentrating in a narrow range from 0.70933 to 0.70959, which corresponds to a sharp peak in a kernel density estimation with a major mode at 0.70943 (Fig 4A; Fig 5). The ^87^Sr/^86^Sr ratios of the adult females ranged between 0.70877 and 0.71186. Their kernel density curve exhibited a wider major mode at 0.70941, while a second accumulation formed a shoulder around 0.71053. The males comprised the highest as well as the lowest ^87^Sr/^86^Sr ratios found at the cemetery. Most of their data concentrated between 0.70946 and 0.71193 with two peaks of the kernel density curve at 0.70981 and 0.71061. Data between 0.70877 and 0.70946 lacked among the males but occurred frequently among the children and the females. The pig teeth and a cattle tooth from the settlement next to the cemetery yielded ^87^Sr/^86^Sr values between 0.70889 and 0.71006 and overlapped completely with the human data (S1B Table). Human enamel, human bones as well as pig and sheep/goat enamel from four Neolithic sites in up to 20 km distance from the cemetery of Mözs exhibited ^87^Sr/^86^Sr ratios that were largely similar to each other and to the range of the animal enamel of the Migration period (0.70879 to 0.71001; n = 32; one sample of human enamel: 0.71062 (S1C Table, Fig 6)).

### Biologically available strontium and local isotope ranges

The Carpathian Basin is largely covered by loess, loam, and alluvial sediments with restricted variation of the isotopic composition of the biologically available strontium, with typical ^87^Sr/^86^Sr ratios between 0.7090 and 0.7105 (48–50, 74–79) (S2 and S3 Figs). At Mözs, two standard deviations over the average of the enamel samples of the pigs and cattle gave a range of 0.70862 to 0.71035. This span covers the isotope ratios of the water of the Danube as well as a modern plant and a soil sample from about 2.5 km away from the site, even though modern anthropogenic influence cannot be excluded at the sampling location [48]. The ^87^Sr/^86^Sr ratios of the animal teeth from Mözs largely represent those of the biologically available strontium in the Carpathian Basin (Fig 6). Within this range, they are more variable than the data spectra of animal and human enamel from many other localities, which implies that they may overestimate the isotopic variation of the bio-available strontium at the site. ^87^Sr/^86^Sr ratios of human and animal teeth and human bones from four chronologically older, Neolithic, sites in a similar geologic setting in up to 20 km distance confirm this impression (Fig 6). These data concentrate between 0.70879 and 0.70952 and overlap completely with the lower half of the data spectrum of the animal teeth of the Migration Period at Mözs. With only two human enamel values being more radiogenic than 0.70952, the consistency of the data among the different sample materials confirms the largely sedentary lifestyle of the Neolithic population with land use strategies concentrating near the settlements. Moreover, despite it cannot be ascertained that any single value of human enamel represents a person who grew up near the site where its remains were found, the overall data distribution appears to provide a robust characterization of the isotope composition of the biologically available strontium in the Sárköz and the Mezőföld areas west of the middle Danube River.

In addition to these different regional datasets, the distribution of the ^87^Sr/^86^Sr ratios of the human enamel from Mözs itself is highly informative [51], especially because the individuals of the age groups infans I and II revealed a peculiar concentration of isotope ratios. At Mözs, 70% of the children (14/20) represent a distinct data range (0.70933–0.70959), which matches the centre of the data spectrum of the animals (Fig 5). It also overlaps with the ^87^Sr/^86^Sr ratios of the Neolithic samples from up to 20 km distance, but does not represent their entire span (Fig 6). Building on this observation, we used the children’s teeth to subdivide the local baseline range at Mözs into three sections: ‘Local Low’ (LL), ‘Local Center’ (LC), and ‘Local High’ (LH). Two standard deviations over the average of the main data accumulation of the children gave a range between 0.70928 and 0.70958, which we ascribed as ‘Local Center’. Adults with enamel values within this range may have grown up on a diet from the same dietary catchment as the children. They possibly represent individuals who were born into the community of Mözs and lived locally into their adult years. ‘Local High’ are ^87^Sr/^86^Sr ratios between the upper end of the ‘Local Center’ spectrum and the upper end of the isotopic range of the animal teeth (0.70958 to 0.71035). Conservatively, we include this span into our estimation of ‘local’ values, even though only one human tooth among the Neolithic samples yielded a value in this range, whereas it did not occur among the animal teeth and human bones from the chronologically older material. ‘Local Low’ is the ^87^Sr/^86^Sr range between the lower end of the ‘Local Center’ spectrum and the lower end of the isotopic range of the animal teeth from Mözs (0.70862 to 0.70928). Sr isotope data within this range occurred frequently among the different categories of Neolithic samples from the wider area (Fig 6). Sr isotope ratios within the LL and LH ranges suggest that the respective individuals did not receive their childhood diets from the very same arable lands that formed the limited data spectrum of the children’s teeth, but from a wider or different home range, possibly similar to what is reflected in the animal teeth. The distribution of the data of the Neolithic samples suggests that the ‘Local low’ section is more representative of the area between the Sárköz and the Mezőföld than the ‘Local high’ range.

Because neither the more conservative nor the more restricted estimates of the local ranges are exclusive to Mözs, non-local individuals may remain unrecognized. Especially, ^87^Sr/^86^Sr ratios in the ‘Local Center’ and ‘Local High’ ranges are typical at sites east and north of the Tisza River in the Eastern Carpathian Basin (S2 Fig). Consequently, non-local individuals from these areas are isotopically indistinguishable at Mözs.

### Data distribution among the burials at Mözs

Sr isotope values in the three parts of the local range and those above or below it occurred in different frequencies among females, males and children, individuals with and without artificial skull deformations, and burials in the three different burial groups (Fig 5, S1A and S1E Table, S4 Fig). Among the 75 individuals, for which Sr isotope data are available, 24 (32%) yielded enamel values outside the local range. Twenty-three of them had ^87^Sr/^86^Sr ratios above and one had an ^87^Sr/^86^Sr ratio below it. The non-local individuals included three (15%) of the 20 children of the age groups infans I and II, ten (33.3%) of the 30 adult females and 11 (45.8%) of the 24 adult males. The ‘Local High’ range occurred in seven (23.3%) of the females and eight (33.3%) of the males. Females (7) dominated the ‘Local Low’ range, which was almost lacking among the data of the children (1) and males (1). In contrast to the dominance of children in the central part of the local range (13; 65%), comparatively few females (6; 20%) and males (4; 17%), as well as one adult of indeterminate sex produced ^87^Sr/^86^Sr ratios in this range.

The isotope ratios of the individuals with artificial skull deformations (n = 45) exhibited a bimodal distribution (Figs 4 and 5). The majority (19; 42%) had ^87^Sr/^86^Sr ratios above the local range, followed by 15 (33.3%) individuals with isotope ratios in the central part of the local spectrum (S1E Table). All of the three children, all of the ten females and six of the ten males with ^87^Sr/^86^Sr ratios above the local range had artificially deformed skulls, and most of these values grouped between about 0.71050 and 0.71100. Among the individuals with skull deformations and ^87^Sr/^86^Sr ratios in the central part of the local range, most were children [11], two were adult females, and one was an adult male. Most individuals with non-modified skulls had ^87^Sr/^86^Sr ratios in one of the local ranges. Non-locals with non-modified skulls comprised four males with ^87^Sr/^86^Sr ratios above and one with an ^87^Sr/^86^Sr ratio below the local range. Most of the females with Sr isotope ratios in the lower part of the local range had non-modified skulls.

With regard to the burial groups, in group A, most individuals had ^87^Sr/^86^Sr ratios in the central and higher part of the local range, while one female had a higher and one male had a lower Sr isotope ratio (S3 Fig; S1E Table). However, these observations need to be treated with caution as not every burial in group A contained adequate skeletal material for sampling. Burial group B revealed representatives of all three parts of the local range and non-local values above it. The non-local individuals included seven adult females, seven adult males, and no children. All individuals with ^87^Sr/^86^Sr ratios in the ‘Local Low’ range belonged to burial group B, with six of them buried next to each other in the main row of this section. Burial group C contained all of the three non-local children with isotope ratios above the local range as well as three non-local males and two non-local females. Six children and none of the adults in group C had ^87^Sr/^86^Sr ratios in the central part of the local range.

### Carbon and nitrogen isotope data

Collagen preservation of the newly analysed C and N isotope samples of human and animal bones was excellent and fulfilled the established quality criteria for archaeological collagen [86, 87] (S1A Table; S1B Table, S1F Table).

**Fig 7:**
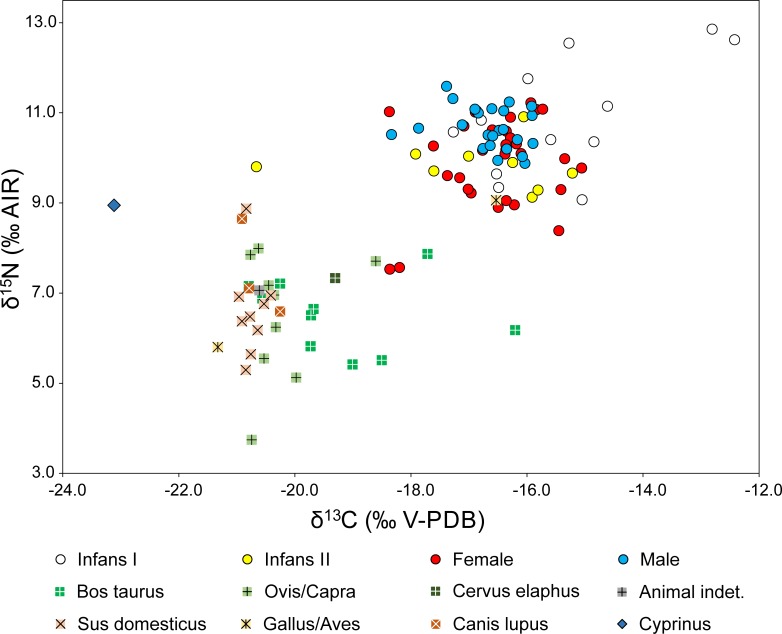
Carbon and nitrogen isotope ratios of human and animal collagen from Mözs. The figure combines the data of this study with those of bone collagen from burial group A and animals from the settlement previously published by Hakenbeck et al. (48).

The carbon isotope ratios of the human samples varied between -20.7 and -12.4 ‰ (Fig 7). The lowest value (grave 70, infant?) and the two highest values (graves 64 and 41, both infans I) were clearly separated from the main data cluster which fell between -18.4 and -14.6 ‰. The nitrogen isotope ratios varied between 7.6 and 12.9 ‰ with the lowest value recorded in a female (grave 22) and the highest value recorded in a child of the age group infans I (grave 41). The females 22 and 67 stand out due to both low δ^13^C and δ^15^N values. Regarding age and sex, the children of the age group infans I had the highest and most variable δ^13^C and δ^15^N values (cf. S1F Table). Both isotope ratios were lower in samples of older children. According to a Mann-Whitney Rank Sum Test, the δ^15^N values of females were significantly lower than those of the males (P < 0.001), whereas the δ^13^C values did not differ significantly between both sexes (P = 0.531). The higher δ^15^N values in males resulted from the fact that 12 of 32 females yielded δ^15^N values below 9.9 ‰, the lowest value found among the males. Considering both isotope ratios in combination, most males formed a comparatively tight data cluster, while many females and older children had lower δ^15^N and more variable δ^13^C ratios (Fig 7).

Regarding the three burial groups, the δ^13^C and the δ^15^N values of the adults overlapped widely and the average values were very similar. Pairwise comparisons of the normally distributed δ^13^C values using Student’s or Welch’s t-tests and not-normally distributed δ^15^N values using Mann-Whitney Rank Sum Tests did not reveal any significant differences (S1F Table). The aforementioned sex-related dissimilarities also existed with regard to the burial groups. The average δ^15^N values of the males were consistently higher than those of the females from the same groups.

Adults with artificially deformed skulls had significantly higher δ^13^C values than adults with non-modified skulls (Welch’s t-test P = 0.0163) (S1F Table, S4 Fig). Nine of the 32 individuals with non-modified skulls had δ^13^C values below -17.2 ‰, the lowest value found among the adults with artificially deformed skulls. Lower average δ^13^C values in individuals with non-modified skulls also existed for both sexes, but were not statistically significant (Student’s t-test females: P = 0.0786; males: P = 0.171). Variation in δ^15^N depended primarily on the sex of the individuals. Differences between individuals with and without artificial skull deformations were not significant.

The δ^13^C values of the animal bones ranged between -23.1 and -16.2 ‰ with the lowest value found in a fish bone (*Cyprinus carpio* [48]) and the highest value found in a cattle bone (Fig 7; S1B Table). The δ^15^N values varied between 3.8 ‰ (sheep/goat) and 9.1 ‰ (chicken (48)). Regarding the species, cattle had higher and more variable δ^13^C values (-20.8 to -16.2; avg.: -19.2 ± 1.4 ‰; n = 10) than sheep/goat (-20.8 to -18.6; avg.: -20.3 ± 0.7 ‰; n = 9) and pigs (-21.0 to -20.4 ‰; avg.: -20.7 ± 0.2 ‰; n = 9). The carbon isotope ratios of cattle point to variable contributions of C_4_ species to a primarily C_3_-plant-based diet. Indication for remarkable shares of C_4_ plants at the base of the food chain was rare for sheep/goat and absent for pigs. The average δ^15^N values were very similar among the three species (cattle: 6.5 ± 0.8 ‰, sheep/goat: 6.5 ± 1.4 ‰; pig: 6.6 ± 1.0 ‰) and do not indicate any significant contribution of meat to the pigs’ diets. Two samples of chicken yielded considerably different isotope ratios, a sample of freshwater fish had a low δ^13^C and a high δ^15^N value, and the isotope ratios of the dog samples were indistinguishable from those of the herbivores.

The average δ^13^C values of -16.6 ± 0.7 ‰ for the adult humans and the combined average of -20.0 ± 1.1 ‰ for cattle, sheep/goat and pig resulted in an average difference of 3.4 ‰ between the fauna and the humans. This is larger than the expected difference of 0 to 2 ‰ between representatives of two adjacent trophic levels in the same food chain [68]. When only considering cattle, the average difference between the animals and humans is still larger than typical for one trophic level (2.6 ‰). The average δ^15^N values were 10.3 ± 0.9 ‰ for the adult humans and 6.5 ± 1.1 ‰ for cattle, sheep/goat and pig, which revealed an average difference of 3.7 ‰. This is within the expected range of one trophic level [71] and could be reached if animal-derived proteins contributed substantially to the human diet. Considering both isotope ratios together, a human diet that consisted largely of meat and dairy products of the cattle with the highest δ^13^C values, would be sufficient to result in the average collagen values of the humans. However, given that most of the animals revealed lower carbon isotope values, this scenario seems unlikely. Instead, the data suggest that other foodstuffs with higher δ^13^C values contributed significantly to the human diet. These dietary items were very likely based on millet, a cereal with C_4_ photosynthesis. Overall, the human data spectrum points to a mixed diet based on typical European C_3_ plants, with considerable contribution of millet and meat and/or dairy products, while fish and poultry may have contributed nitrogen with high δ^15^N levels.

## Discussion

The cemetery of Mözs was probably founded around 430/440 AD and used by two or three generations until about 470/480 AD. All datasets attest to a remarkably heterogeneous community. Especially the strontium isotope ratios were considerably more variable than at prehistoric sites in the geologically homogeneous Carpathian Basin (S2 Fig). They indicate that most of the adult population changed their places of residency at least once in life. Thereby Mözs contributes to an emerging picture of considerable human mobility in the area during the 5^th^ and 6^th^ century [48, 49]. In combination with each other, the strontium and light stable isotope data, artificial skull deformations, burial types, and grave goods reveal several groups of people that formed the multifaceted community of the site and have strong implications for cultural and population changes and amalgamation after the decline of the Roman Empire.

### The founder generation

Archaeological evidence identifies at least two early centres of the cemetery: one in group A [graves 23, 11], and another in the centre of the main row of group B [brick structure graves: 54, 57, 58, 60, 61] (Fig 1). Archaeologically, the brick-lined graves as well as the small number or lack of grave goods (*reduzierte Beigabensitte*) could be understood as late Antique traditions [88]. The strontium isotope ratios of the supposed members of the founder generation spanned across all three sections of the local range (Fig 5; S4 Fig). Their δ^13^C values of between c. -18 ‰ (graves 60, 54) and -16 ‰ (graves 58) attest to variable contributions of millet to their average diets (S5 Fig). The δ^15^N values were comparatively high. Only one female (grave 57) had a δ^15^N value below the lowest value of the males, whereas such values occurred much more often among the later dating females. Grave 11 stands in contrast to the inhumations in late Antique tradition. This unique North-South-oriented burial lies c. 10–15 m west of group A, making its connection to the whole cemetery problematic. While its grave goods suggest an early, mid-5th century dating, it is probably one of the earliest burials at the site. Its grave goods including food offerings in form of a jar and animal bones attest to a different cultural tradition and indicate strong connections to the solitary burials of the Hun period [7, 89–91]. If we consider the individual in Grave 11 to be a part of the community, then the founder generation at Mözs likely included representatives of the late Roman population groups and possibly Hunnic period migrants.

### A non-local group of similar isotopic and cultural background

**Fig 8:**
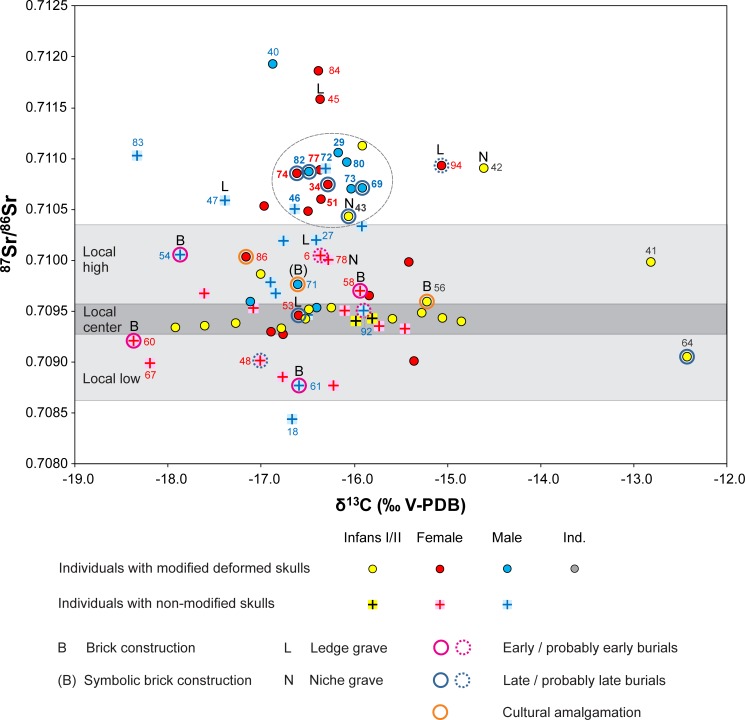
Scatter plot of δ^13^C values of bone collagen and ^87^Sr/^86^Sr ratios of enamel of the same individuals at the cemetery of Mözs. The grey ellipse highlights a tight cluster of individuals with highly similar Sr and C isotope ratios. Bold burial numbers indicate individuals who exhibit also very similar δ^15^N values.

Combined evidence of the isotope, archaeological, and anthropological data identified twelve individuals of supposedly shared origin and cultural tradition [graves 29, 34, 43, 46, 51, 69, 72, 73, 74, 77, 80, 82]. They had radiogenic strontium isotope ratios above the local range (c. 0.7105–0.7110), and similar δ^13^C (-16.6 to -15.9 ‰) and δ^15^N (9.8 and 11.2 ‰) values (Figs 5 and 8; S4 Fig and S5 Fig). The isotopically similar individuals also comprised five of the nine burials with indications of a later date. This suggests that this group joined the already existing community about a decade after the establishment of the cemetery. Nine of these burials expanded the previously established grave rows in burial group B and three were probably among the founders of burial group C (Fig 1). The cluster of individuals lacked inhumations of burial group A (S4 Fig) as well as burials with archaeological evidence for an early date. Ten of the twelve burials (5 males, 4 females, 1 child) had artificially deformed skulls. Both individuals with non-modified skulls were male. Five inhumations in ledge or niche graves–including four with an artificially deformed skull–also had radiogenic, non-local ^87^Sr/^86^Sr ratios, but at least one of the light stable isotope ratios outside the data ranges of the core group. A prime example for the later arriving individuals is the child burial in the niche grave 43 (Fig 2), an archaeologically determined girl with an artificially deformed skull (S1 Fig) and exceptionally rich grave goods that date to the middle of the 5^th^ century. Very rich burials were also common among inhumations of the first generation at other cemeteries indicating the presence of overrepresentation as a result of social competition among the members of newly formed or changing communities [92].

Especially the high number of children with artificially deformed skulls and strontium isotope ratios in the centre of the local range suggests that the arriving group became culturally and socially dominant at the cemetery. They retained the custom of shaping the bodies of their offspring irreversibly, and artificial skull deformation became an integral part of the community’s identity [93, 94]. While artificial skull modification has already been practiced in the Carpathian Basin during the 4^th^ century, the practise flourished especially during the middle third of the 5^th^ century, when it occurred frequently among both males and females [95]. The widespread re-emergence of this tradition is clearly related to newly founded, small-sized burial sites that were mostly established by foreign, non-Roman population groups, while individuals with intentionally modified skulls are rarely present in the continuously used late Roman cemeteries. The high frequency of skull deformation among children at Mözs stands in contrast to contemporary cemeteries in areas such as southern Germany. At these sites, this phenomenon only occurred among adult individuals, most of them being females [93, 96].

### Amalgamation of late antique and ‘Barbarian’ traditions

The co-habitation of people with different cultural backgrounds also facilitated an amalgamation of local and foreign traditions in the same burials. The brick-lined structure of Grave 56 represents a late Antique tradition, while the artificially deformed skull of the locally born child buried in it is characteristic for the newly arriving group. A possible interpretation is that the members of the founding generation already practised artificial skull deformation. However as this would be the only example of an earlier dating of this tradition, the explanation of cultural amalgamation is more plausible. While the ‘fashion’ of artificial skull deformation became popular in an instant, burial customs may have changed slower and retained their local character during the transition. Moreover, the age (7–8 years old) of the individual buried in Grave 56 presents a chronological problem: subsequent generations could have the same archaeological dating, because children (especially very young ones) may have died before their parents. Another example is grave 71, an adult male with a deformed skull and few bricks placed in his grave. As *pars pro toto*, these could be understood as a symbolic brick structure, a later, simplified version of the construction and illustrate the hybridization of different strands of traditions. Grave 86, the burial of a juvenile female, shows that local artefact types, in this case late Roman earrings, could also appear together with artificial skull deformation. Reversely, there is no example of foreign artefact types in brick-lined graves, which usually contained very few or no grave goods and disappeared shortly after the arrival of the foreign group.

Members of the mixed generation mostly appeared in the burial groups B and C. Group C consisted of children and adults with ^87^Sr/^86^Sr ratios above or in the upper section of the local range as well as children with isotope ratios in the restricted central part of the local range (S4 Fig). This implies that burial group C was used by first-generation non-locals and their offspring who died as children. The burial group was probably abandoned before locally born members of the second generation died in their adult years. A similar pattern appeared at the cemetery of Szólád near Lake Balaton that was supposedly used by only one generation during the Lombard period in the 6^th^ century [49].

### Interaction with other contemporary groups

Neither the individuals who primarily exhibited late Antique traditions nor the non-local individuals who practiced artificial skull deformation have necessarily arrived at Mözs in single events. Instead, connections with the former homelands and movement of individuals in both directions may have persisted during the whole period of use of the site. Moreover, the data structure indicates a likely flow of migrants in and out of the community. Children were much more abundant among the individuals in the centre of the local ^87^Sr/^86^Sr range. This indicates that only few individuals who grew up at Mözs also died at this place as adults. Because the archaeological record suggests that the burial site was in use for about 30 to 40 years, it is very likely that a remarkable number of people, who lived beyond childhood, left the community at any stage of their lives and were buried elsewhere. There is no indication of a strongly sex-biased exogamy away from Mözs, and the supposed emigrants likely included single males and females, small groups, or families.

Furthermore, persons of various places of origin have likely joined the community–possibly even before the founder generation and the later arriving and amalgamating groups reached the site of Mözs. Examples are the individuals 40, 45 and 84 with Sr isotope ratios above 0.7115, or the male in grave 18 with the lowest ^87^Sr/^86^Sr ratio (Fig 5). The individuals in the lower section of the local range, most of them females with non-modified skulls in burial group B, may also go back to a distinct area of origin, supposedly not in the eastern half of the Carpathian Basin. They may have originated from the near vicinity of the cemetery as this range of ^87^Sr/^86^Sr values is frequently documented among Neolithic human and animal samples from four sites in up to 20 km distance (Figs 1 and 6, S2 and S3 Figs). Unlike stated for the potentially emigrating individuals, the dominance of females in the lower Sr isotope spectrum may indeed reflect exogamous marriage practises similar to what isotope data imply for earlier prehistoric periods [32].

### Implications of the dietary habits

The δ^13^C values of the burials from Mözs were higher than those from previously investigated Early Neolithic to Early Bronze Age sites (c. 6500–1400 BC) in the Carpathian Basin [97, 98]. This result attests to remarkable contributions of millet to the human diet, a C_4_ plant, which first started to play a role in the every-day diet in the area during the Late Bronze Age and Early Iron Age [97, 98]. Ancient agrarian texts, archaeobotanical evidence, and stable isotope data state that millet was also cultivated during the Roman period. However–with variation across regions and social groups–it was an overall less important crop than C_3_ cereals, such as barley, wheat, and rye [99, 100]. This observation also applies to the Carpathian Basin, where archaeobotanical investigations documented that C_3_ cereals exceeded millet by far [101]. There is little botanical evidence for the 5^th^ century. However, written sources suggest that the upheavals at the end of the Roman Empire also triggered an increase of millet cultivation in association with a nomadic lifestyle of incoming ‘Barbarian’ people [101]. Alternatively, the collapse of the villa-dominated late Roman rural economy with its well-organized agricultural structures may have caused a shift from the more sensitive wheat-based economy to less demanding millet cultivation.

Previous isotopic evidence stated that millet consumption was generally common in the Carpathian Basin during the 5^th^ century AD, and the burials from Mözs revealed the highest average δ^13^C values among five roughly contemporary sites [48] (S7 Fig). The differences were significant in three of four cases of pairwise comparison, whereas there were no statistically significant differences among the domestic herbivores and pigs (ANOVA; S1F Table). This pattern attests to similar environmental conditions across the Carpathian Basin and isotopic composition of potential animal-derived foodstuffs, so that the higher average δ^13^C values of the burials at Mözs may indeed be ascribed to higher shares of millet in their diet. The data distribution within the burial community suggests that individuals with non-modified skulls and those who were buried in brick-lined graves consumed less millet than those with artificial skull deformations, the supposedly incoming group with non-local traditions (S5 Fig). This observation supports the postulated influence from the Eurasian steppes and relocation of people with shared cultural background and dietary habits. Millet consumption itself, however, does not *per se* indicate nomadic lifestyle practices.

## Conclusion

Archaeological, anthropological, and stable isotope analyses of almost 100 individuals from the cemetery of Mözs demonstrated the coalescence of people and their cultural traditions. The site represents the transition period after the decline of the Roman Empire through the rise and fall of the Hunnic power and ends before the formation of ‘Barbarian’ kingdoms in the Carpathian Basin (430/440-470/480 AD). The community had a very recipient habitus. It accepted and integrated men, women, and children of different geographical and cultural backgrounds during the two to three generations of its existence. The isotope data indicate that residential changes played a remarkable role and occurred not only on an individual basis, but also in groups of a shared cultural background and lifestyle. The founder generation of the cemetery predominantly comprised people, who represented late Antique traditions, grew up in different locations and consumed a diet with variable shares of millet. Little later, a group from a shared area of origin and with similar dietary habits that included millet consumption mixed in. They exhibited foreign, probably non-Roman traditions, among which artificial skull deformation was the most remarkable. While the cemetery grew, both local and these non-local traditions even amalgamated in the same burials. At Mözs, archaeological and bioarchaeological data attest to the 5^th^ century being a highly dynamic time in the Carpathian Basin. Residential changes were widely common and provided the basis for cultural hybridization at the site and even personal level. Placed into the historical narrative, this could be understood as the emergence of a Roman-‘Barbarian’ *Mischkultur* (mixed culture) [2], in which Romanized ‘Barbarians’ and ‘barbarized’ late Roman population groups were indistinguishable.

## Supporting information

S1 Table Data table. **S1A Table.** Anthropological, archaeological and isotope data of the burials from the cemetery of Mözs as well as their classification into Sr isotope data ranges and interpreted group affiliations. **S1B Table.** Results of carbon, nitrogen and strontium isotope analysis of animal bones and teeth from Mözs. **S1C Table.** Strontium isotope data of human teeth and bones as well as animal teeth of Neolithic sites in up to 20 km distance from Mözs. **S1D Table.** Carbon and nitrogen isotope ratios of the quality control standards analysed along with the collagen samples from Mözs. **S1E Table.** Summary counts and percentages of individuals with ^87^Sr/^86^Sr ratios in the different local and non-local isotope ranges according to age and sex, burial groups, and skull deformations. **S1F Table.** Descriptive statistics of C and N isotope data of various groups of individuals at the cemetery of Mözs, results of pairwise comparisons among these groups and of Mözs with other datasets of the 5th century AD.
(XLSX)

S1 Fig Examples of artificially deformed skulls from the cemetery of Mözs. The skulls from grave 34 and grave 43 represent examples of an adult female and an infans II child with very similar deformations in an upward-backward direction and recessions resulting from binding (deformation variant II). The skull of grave 85 (infans I) combines an artificial deformation of variant I (circular binding and strong upward deformation) with post-mortem deformation.
(PDF)

S2 Fig Boxplots of the ^87^Sr/^86^Sr ratios of enamel samples from children, adults and animals from Mözs in comparison to published data from the Carpathian Basin (48–50, 74–79). The sites are grouped according to landscapes as mapped in S3 Fig. See text for definition of the local Sr isotope ranges at Mözs (LH = Local high; LC = Local central; LL = Local low).
(PDF)

S3 Fig Location of sites with published ^87^Sr/^86^Sr data in the Carpathian Basin and areas as distinguished in S2 Fig. For the legend of the geological map, please refer to: ttps://map.mbfsz.gov.hu/fdt_alapszelvenyek/. WMS server of geological map: https://map.mbfsz.gov.hu/arcgis/services/fdt100/fdt_100/MapServer/WMSServer.
(PDF)

S4 Fig Distribution of the ^87^Sr/^86^Sr ratios of enamel of the burials of the cemetery of Mözs. The individuals are grouped according to burial groups A, B and C, and within these groups by subadult individuals as well as adult females and males. Within each group, the data are sorted from lower to higher values. See text for definition of the local strontium isotope ranges.
(PDF)

S5 Fig Carbon and nitrogen isotope ratios of the adult burials at Mözs. The data are grouped according to sex and the presence or absence of artificial skull modifications. Representatives of the supposed founder generation (*), indication of inhumation in the 3^rd^ quarter of the 5^th^ century (°), and individuals with similar C, N and Sr isotope data (‘) are labelled.
(PDF)

S6 Fig Scatter plot of δ^15^N values of bone collagen and ^87^Sr/^86^Sr ratios of enamel of the same individuals at the cemetery of Mözs. The grey ellipse highlights a tight cluster of individuals with highly similar Sr and N isotope ratios. Bold burial numbers indicate individuals who exhibit also very similar δ^13^C values.
(PDF)

S7 Fig Carbon and nitrogen isotope ratios of the adult individuals and animals from Mözs in comparison to contemporary datasets as published in (48). While there are no significant differences among the animal data, the human bones from Mözs yielded the highest average δ^13^C values.
(PDF)
