# Supplementary figures and images for: Coalescing traditions—Coalescing people: Community formation in Pannonia after the decline of the Roman Empire
Source: PLoS One. 2020 Apr 29;15(4):e0231760. doi: 10.1371/journal.pone.0231760 (PMC7190109; doi:10.1371/journal.pone.0231760)

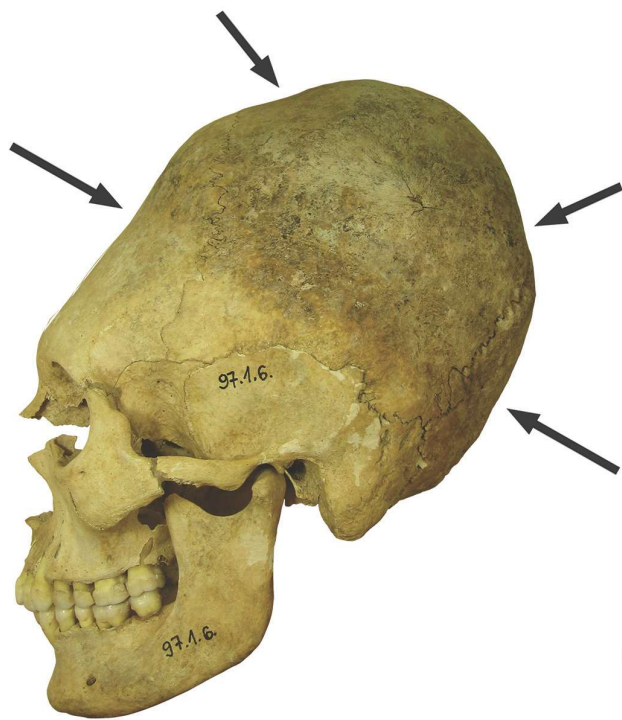

*Grave 34*

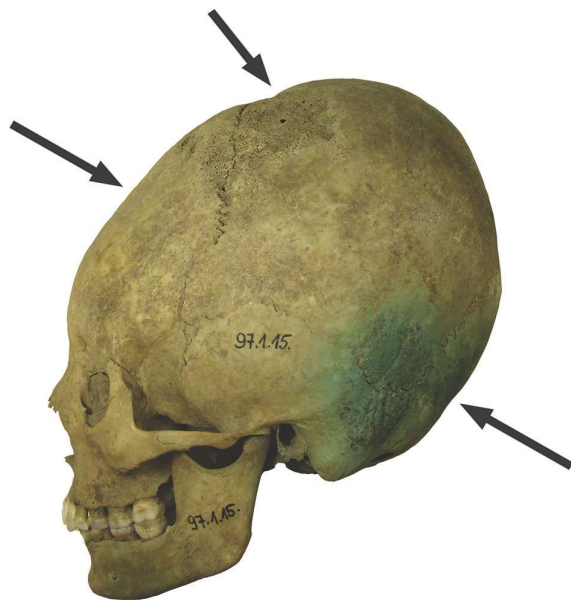

*Grave 43*

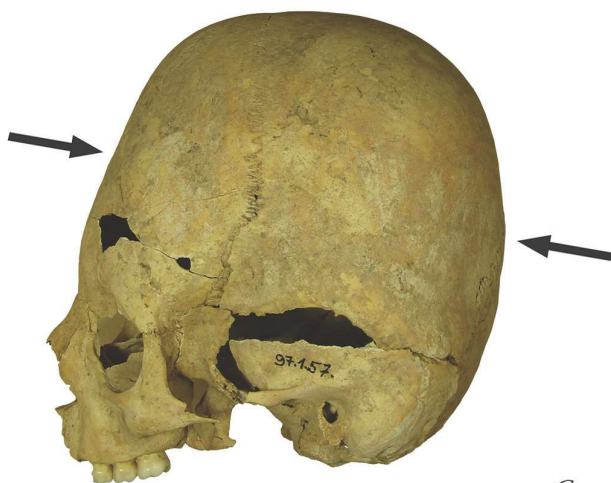

*Grave 85*

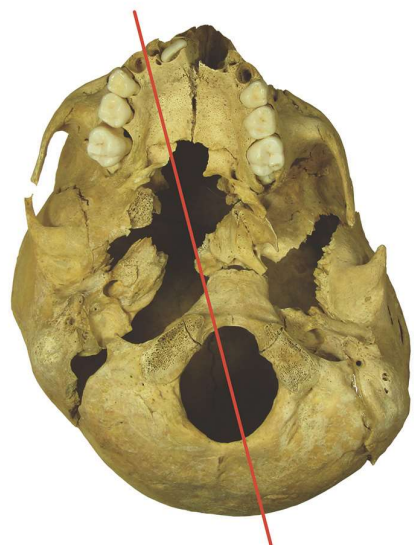

Supplement: S1 Fig — The skulls from grave 34 and grave 43 represent examples of an adult female and an infans II child with very similar deformations in an upward-backward direction and recessions resulting from binding (deformation variant II). The skull of grave 85 (infans I) combines an artificial deformation of variant I (circular binding and strong upward deformation) with post-mortem deformation. (PDF) [file pone.0231760.s002.pdf]

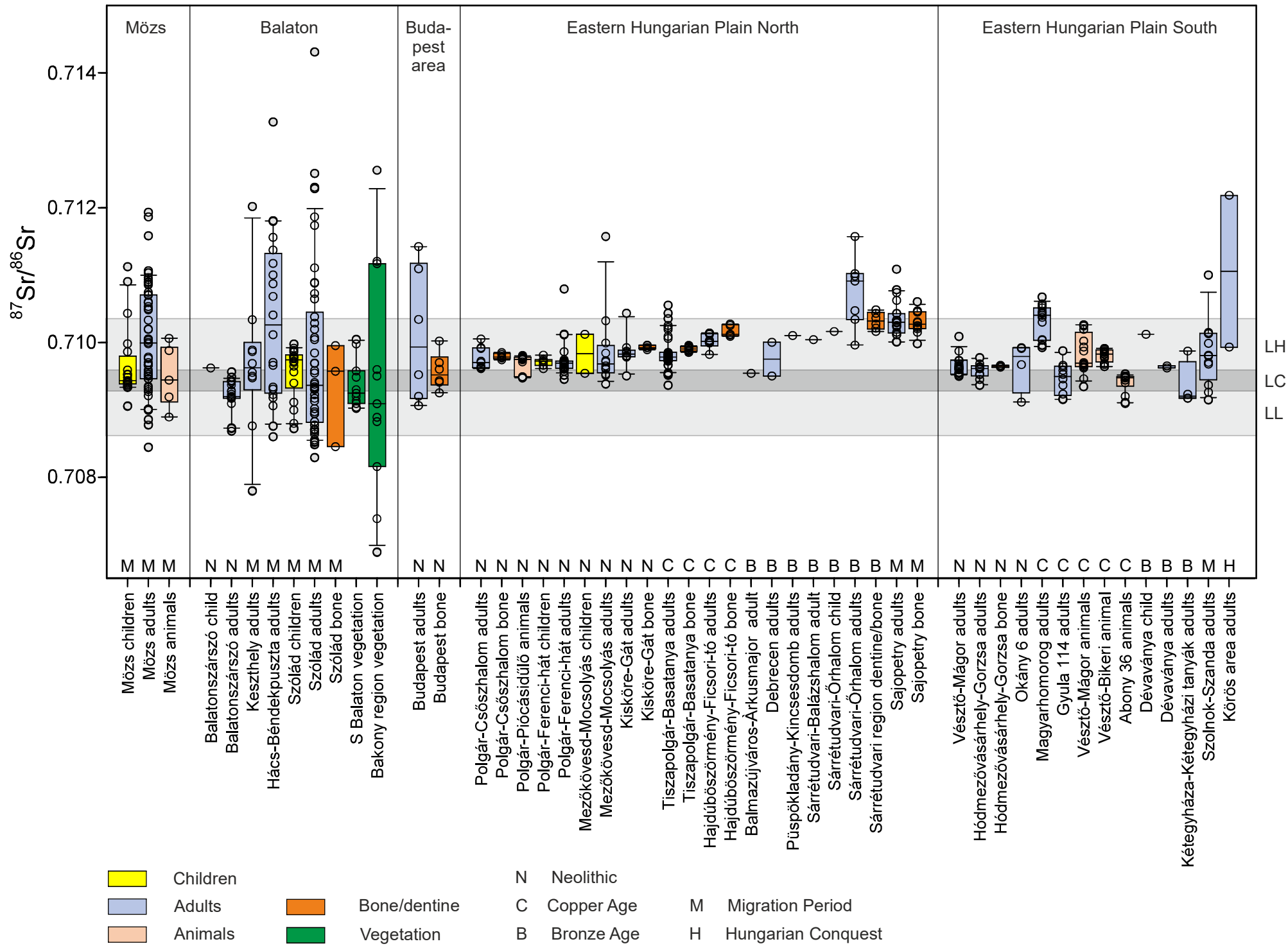

Supplement: S2 Fig — The sites are grouped according to landscapes as mapped in S3 Fig. See text for definition of the local Sr isotope ranges at Mözs (LH = Local high; LC = Local central; LL = Local low). (PDF) [file pone.0231760.s003.pdf]

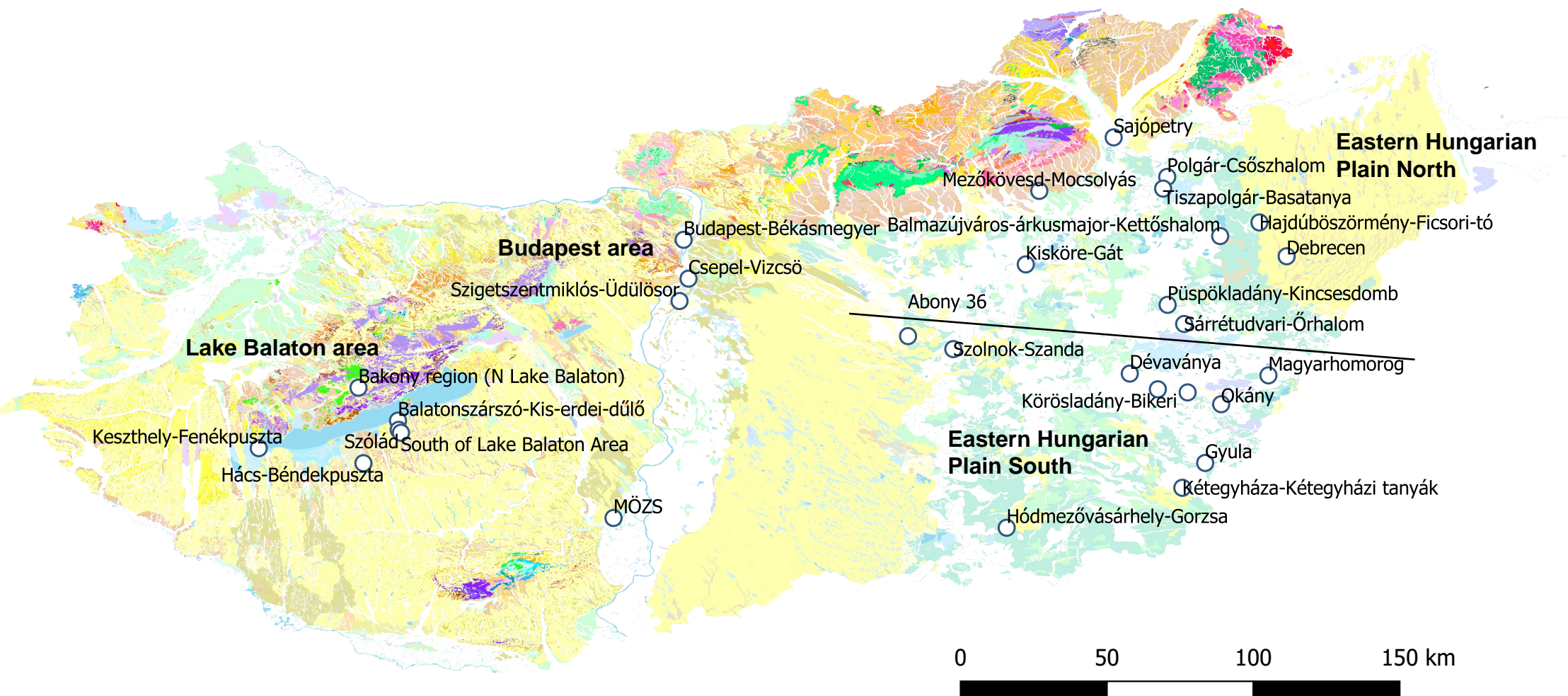

Supplement: S3 Fig — For the legend of the geological map, please refer to: ttps://map.mbfsz.gov.hu/fdt_alapszelvenyek/. WMS server of geological map: https://map.mbfsz.gov.hu/arcgis/services/fdt100/fdt_100/MapServer/WMSServer. (PDF) [file pone.0231760.s004.pdf]

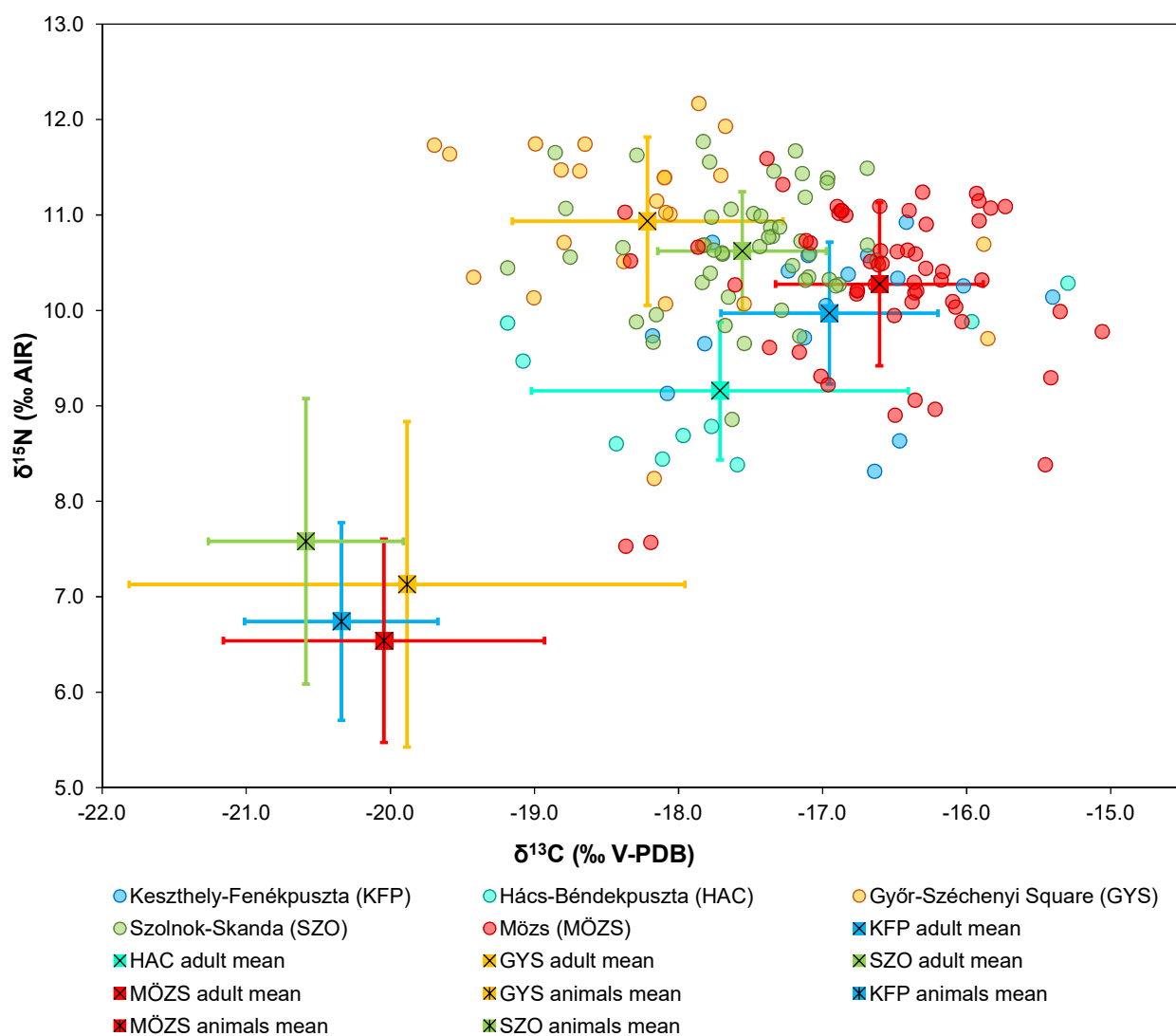

Supplement: S7 Fig — While there are no significant differences among the animal data, the human bones from Mözs yielded the highest average δ13C values. (PDF) [file pone.0231760.s008.pdf]
