# Supplementary material for: Coalescing traditions—Coalescing people: Community formation in Pannonia after the decline of the Roman Empire
Source: PLoS One. 2020 Apr 29;15(4):e0231760. doi: 10.1371/journal.pone.0231760 (PMC7190109; doi:10.1371/journal.pone.0231760)
Supplement: S4 Fig — The individuals are grouped according to burial groups A, B and C, and within these groups by subadult individuals as well as adult females and males. Within each group, the data are sorted from lower to higher values. See text for definition of the local strontium isotope ranges. (PDF) [file pone.0231760.s005.pdf]

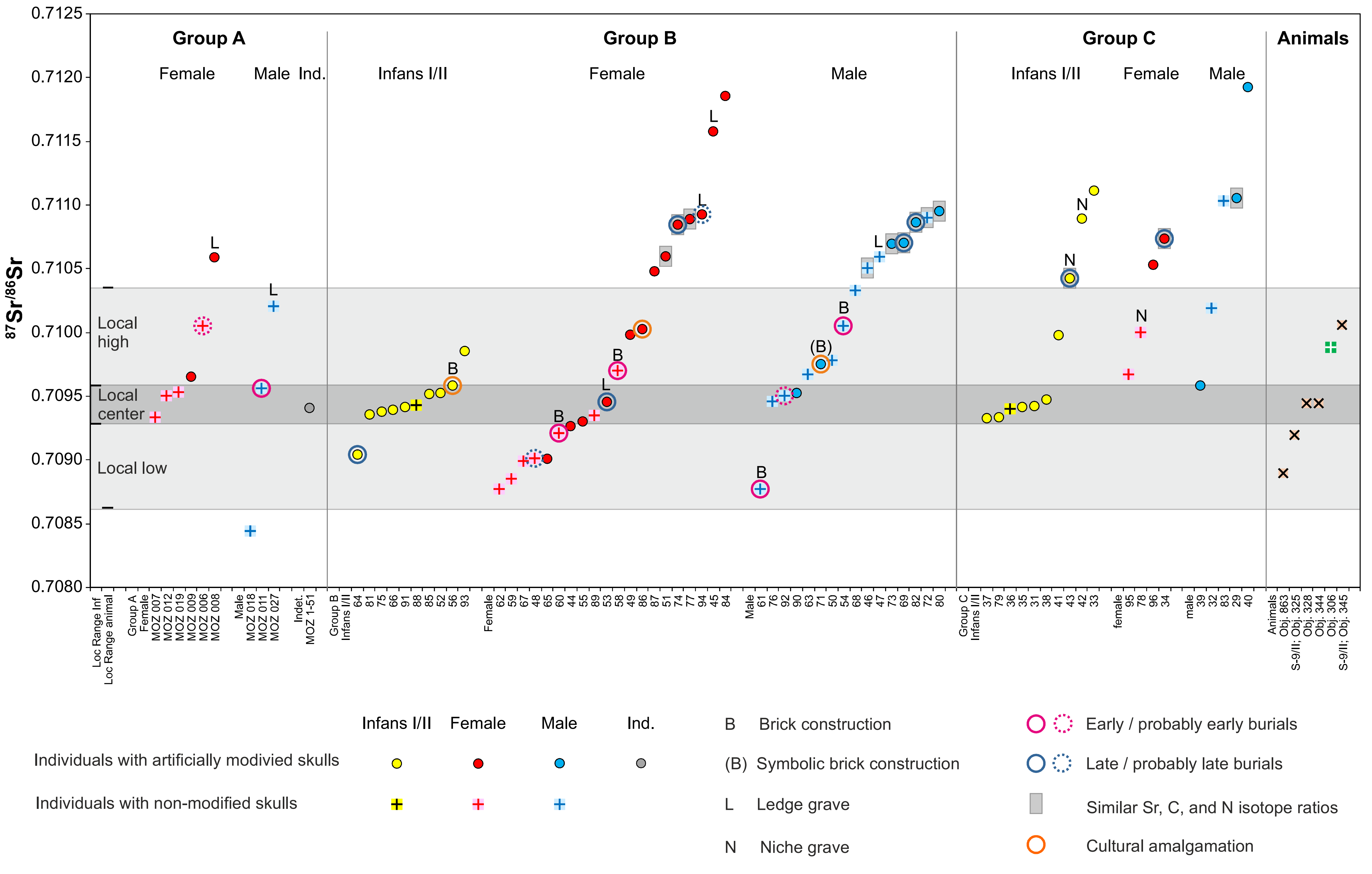

Group A
Group B
Group C
Animals
Female
Male
Ind.
Infans I/II
Female
Male
Infans I/II
Female
Male
$^{87}Sr/^{86}Sr$
0.7125
0.7120
0.7115
0.7110
0.7105
0.7100
0.7095
0.7090
0.7085
0.7080
Local high
Local center
Local low
Loc Range Inf
Loc Range animal
Group A Female
MOZ 007
MOZ 012
MOZ 019
MOZ 009
MOZ 006
MOZ 008
Male
MOZ 018
MOZ 011
MOZ 027
Indet.
MOZ 1-51
Group B Infans I/II
64 81 75 66 91 88 85 52 56 93
Female
62 59 67 48 65 60 44 55 89 53 58 49 86 87 51 74 77 94 45 84
Male
61 76 92 90 63 71 50 54 68 46 47 73 69 82 72 80
Group C Infans I/II
37 79 36 35 31 38 41 43 42 33
female
95 78 96 34
male
39 32 83 29 40
Animals
Obj. 863
S-9/II; Obj. 325
Obj. 328
Obj. 344
Obj. 306
S-9/II; Obj. 345
Infans I/II
Female
Male
Ind.
Individuals with artificially modivied skulls
Individuals with non-modified skulls
B Brick construction
(B) Symbolic brick construction
L Ledge grave
N Niche grave
Early / probably early burials
Late / probably late burials
Similar Sr, C, and N isotope ratios
Cultural amalgamation
