# Supplementary material for: Coalescing traditions—Coalescing people: Community formation in Pannonia after the decline of the Roman Empire
Source: PLoS One. 2020 Apr 29;15(4):e0231760. doi: 10.1371/journal.pone.0231760 (PMC7190109; doi:10.1371/journal.pone.0231760)
Supplement: S5 Fig — The data are grouped according to sex and the presence or absence of artificial skull modifications. Representatives of the supposed founder generation (*), indication of inhumation in the 3rd quarter of the 5th century (°), and individuals with similar C, N and Sr isotope data (‘) are labelled. (PDF) [file pone.0231760.s006.pdf]

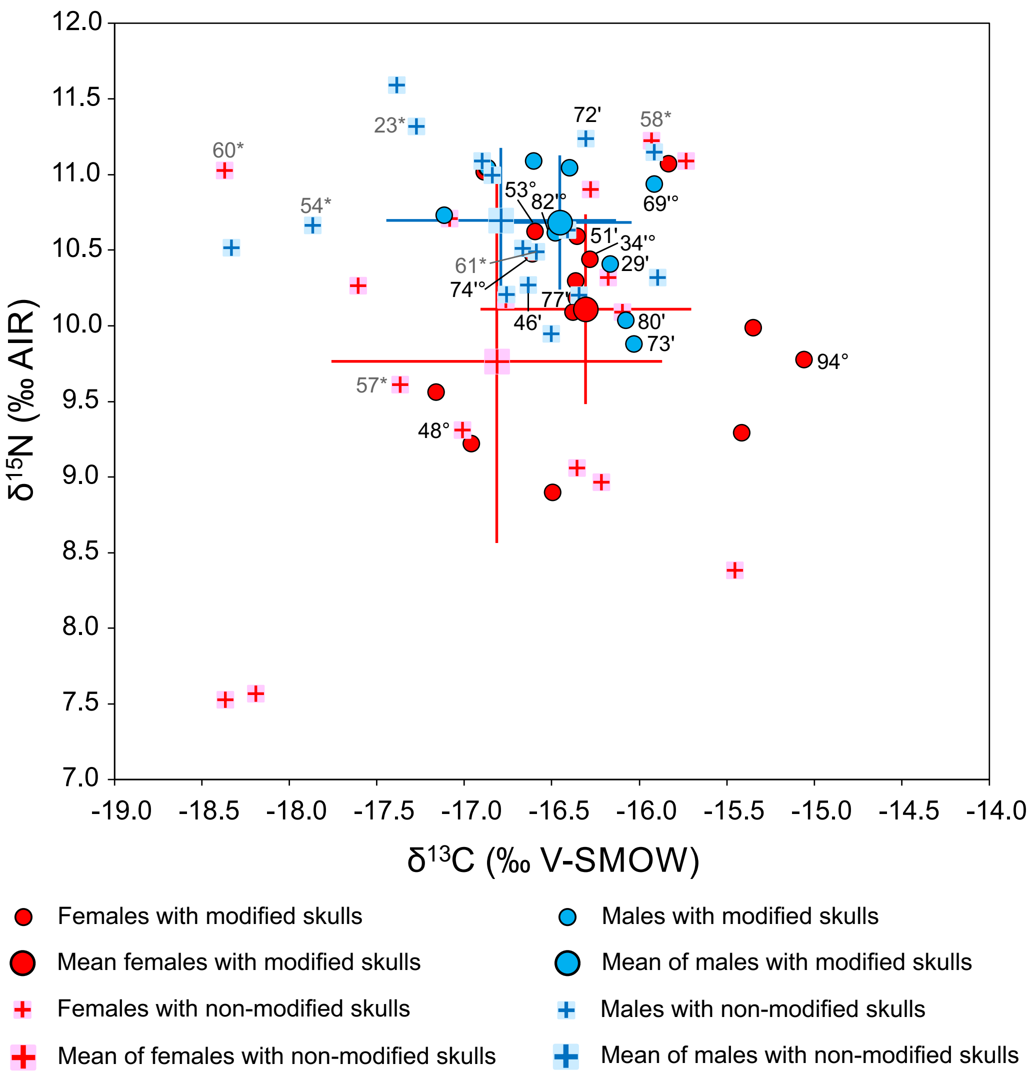

δ15N (‰ AIR)
δ13C (‰ V-SMOW)
12.0
11.5
11.0
10.5
10.0
9.5
9.0
8.5
8.0
7.5
7.0
-19.0
-18.5
-18.0
-17.5
-17.0
-16.5
-16.0
-15.5
-15.0
-14.5
-14.0
23*
72'
58*
60*
54*
53°
82'°
69'°
51'
34'°
29'
61*
74'°
77'
46'
80'
73'
94°
57*
48°
Females with modified skulls
Mean females with modified skulls
Females with non-modified skulls
Mean of females with non-modified skulls
Males with modified skulls
Mean of males with modified skulls
Males with non-modified skulls
Mean of males with non-modified skulls
