# Supplementary material for: Coalescing traditions—Coalescing people: Community formation in Pannonia after the decline of the Roman Empire
Source: PLoS One. 2020 Apr 29;15(4):e0231760. doi: 10.1371/journal.pone.0231760 (PMC7190109; doi:10.1371/journal.pone.0231760)
Supplement: S6 Fig — The grey ellipse highlights a tight cluster of individuals with highly similar Sr and N isotope ratios. Bold burial numbers indicate individuals who exhibit also very similar δ13C values. (PDF) [file pone.0231760.s007.pdf]

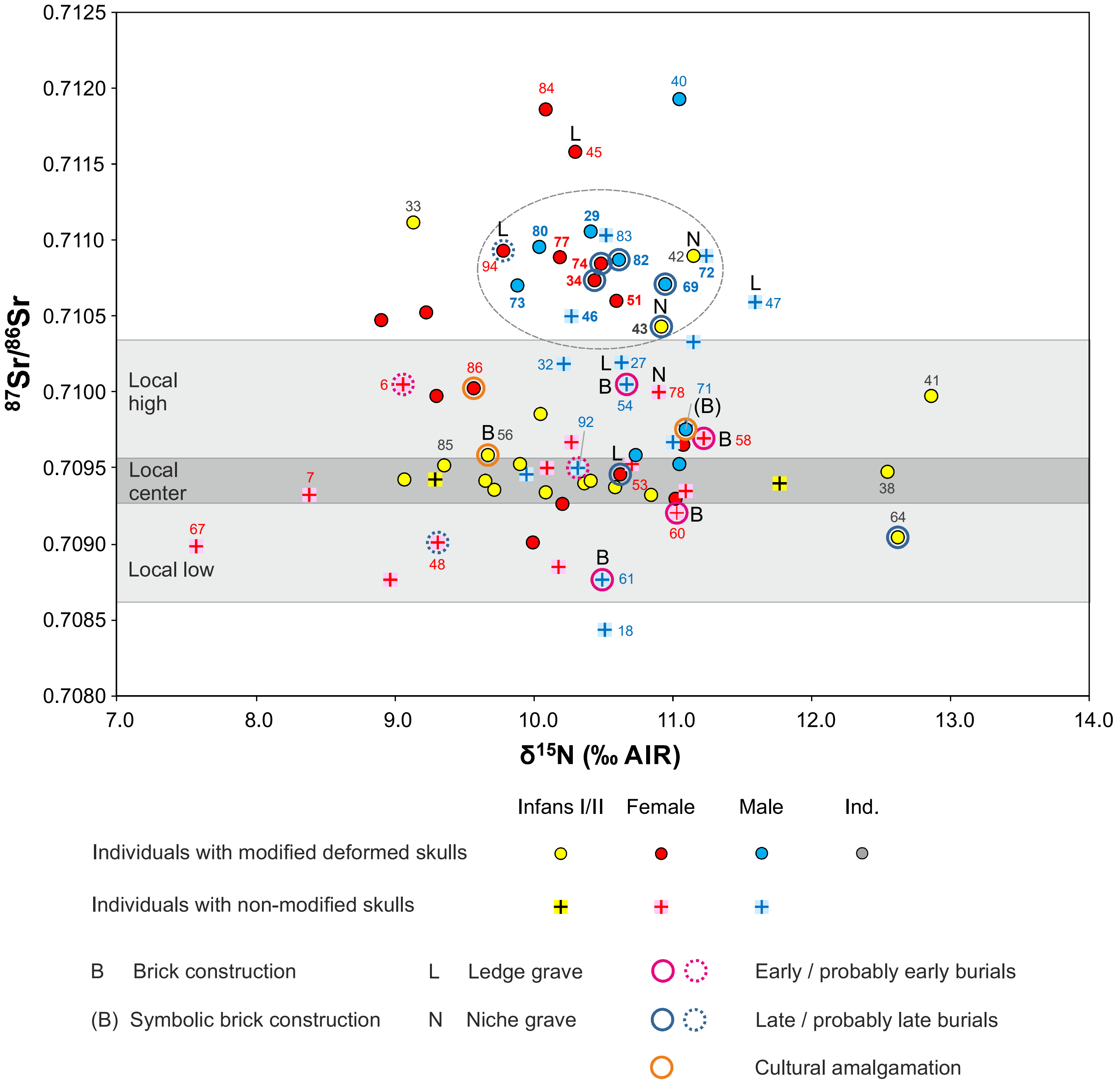

0.7125
0.7120
0.7115
0.7110
0.7105
0.7100
0.7095
0.7090
0.7085
0.7080
$^{87}Sr/^{86}Sr$
7.0
8.0
9.0
10.0
11.0
12.0
13.0
14.0
$\delta^{15}N$ (‰ AIR)
Local high
Local center
Local low
84
40
L 45
33
29
80
L
77
83
N
42
94
74
82
72
34
69
L
73
51
47
46
N
43
32
L 27
86
N
6
B
71
54
(B)
41
92
B 56
B 58
85
L
7
53
38
B
60
64
67
48
B
61
18
Infans I/II
Female
Male
Ind.
Individuals with modified deformed skulls
Individuals with non-modified skulls
B Brick construction
L Ledge grave
Early / probably early burials
(B) Symbolic brick construction
N Niche grave
Late / probably late burials
Cultural amalgamation
